# Inconsistent Time-Dependent Effects of Tetramethylpyrazine on Primary Neurological Disorders and Psychiatric Comorbidities

**DOI:** 10.3389/fphar.2021.708517

**Published:** 2021-08-20

**Authors:** Yue-Peng Jiang, Yan Jin, Jie Bao, Song Wang, Wei-Dong Lai, Cheng-Ping Wen, Zheng-Hao Xu, Jie Yu

**Affiliations:** ^1^ College of Basic Medical Science, Zhejiang Chinese Medical University, Hangzhou, China; ^2^ Second Affiliated Hospital, Zhejiang Chinese Medical University, Hangzhou, China; ^3^ Key Laboratory of Neuropharmacology and Translational Medicine of Zhejiang Province, Hangzhou, China

**Keywords:** epilepsy, chronic pain, tetramethylpyrazine, neuropsychiatric comorbidities, time dependent effects

## Abstract

The aim of this study was to investigate the time dependent effects of tetramethylpyrazine (TMP, main activity compound of *Ligusticum chuanxiong* Hort) on two neurological disorders and their neuropsychiatric comorbidities. 6 Hz corneal rapid kindling was used to induce epileptogenesis and the inflammatory pain was induced by intra-articular Complete Freund’s adjuvant (CFA) injection. The mechanical pain thresholds were measured using von Frey hair (D4, D11, D18, D25 after CFA first injection), and the vertical rearings of the mice was observed. To test the neuropsychiatric comorbidities, anxiety-like behaviors of mice were examined by open field and elevated plus maze tests. Two behavioral despair models, tail suspension test and forced swimming test were also used to evaluate the depressive like behaviors. The results showed that TMP administered from the initial day (D1-D35 in kindling model, D0-D14 and D0-D28 in CFA model) of modeling retarded both the developments of 6 Hz corneal rapid kindling epileptogenesis and the CFA induced inflammatory pain. In comparison, late periods administration of TMP (D21-D35 in kindling and D14-D28 in CFA model) showed no effect on the epileptogenesis and the generalized seizures (GS) of kindling, but alleviated maintenance of CFA induced inflammatory pain. Furthermore, we also found all TMP treatments from the initial day of modeling alleviated the co-morbid depressive and anxiety-like behaviors in both models; however, late periods treatments did not, either in kindling or the CFA induced inflammatory pain. BDNF/ERK signaling impairment was also tested by western blot, and the results showed that TMP administered from the initial day of modeling increased the hippocampal BDNF/ERK expression, whereas late period administration showed no effects. Overall, our findings reveal the inconsistent time dependent effects of Tetramethylpyrazine on neurological disorders and their relative neuropsychiatric comorbidities, and provide novel insight into the early application of TMP that might enhance hippocampal BDNF/ERK signaling to alleviate neuropsychiatric comorbidities in neurological diseases.

## Introduction

Depression, anxiety, and other psychiatric disorders have been widely reported as pervasive persisted neuropsychiatric comorbidities that were commonly diagnosed in patients with neurological diseases (Chiang and Cheng 2014). For example, depression is the most common comorbidity among patients with drug-resistant temporal lobe epilepsy (TLE) (D'Alessio et al., 2020). Depressive or anxiety disorders are also common occurrence in about ∼50% of patients with chronic pain (Juang et al., 2000; Gerrits et al., 2014). 23.1% of the Public Safety Personnel (PSP; e.g., correctional service officers, firefighters, police officers) respondents suffering from chronic pain were self-reported clinically posttraumatic stress disorder (PTSD), major depressive disorder, generalized anxiety disorder or social anxiety disorder (Carleton et al., 2018). As psychiatric disorders are always associated with disrupted sleep, fatigue, loss of psychomotor activity and reduced energy, they usually contribute high privilege of suicide in clinic (Mann et al., 1999; Knipe et al., 2019). Additionally, depression and anxiety have also been consistently considered as the crucial causes of opioids accidental abuse and misuse (Moffat et al., 2019; Volkow et al., 2019). There is mounting evidence also showing a bi-directional association that patients, with psychiatric disorders history, were more likely to have a higher risk of neurological disease (Kanner et al., 2018). Though high rates of neuropsychiatric comorbidities were observed in clinic, the pathogenesis of co-morbid psychiatric disorders in neurological disease is elusive. Thus, comprehensive insights are needed by studying psychiatric disorders (as co-morbid problems) in neurological disease.

Epilepsy is one of the most privileged neurological diseases with serious mental illness complications (Beletsky and Mirsattari 2012). Six percent of people with epilepsy in the general population appear to suffer from a psychiatric disorder, while it rises to ∼20% in populations with temporal lobe and/or refractory epilepsy (Gaitatzis et al., 2004). Some of the anti-epileptic drugs, such as barbiturates, vigabatrin and topiramate, also show greater associations with depressive comorbidities, presenting in up to 10% of all patients, and even more so in susceptible patients (Mula and Sander 2007). So, improvement of life quality might depend more on treating comorbidities than seizures in refractory epilepsy patients. There is also an unmet need of novel pharmacotherapies that could both prevent the epileptic seizure and alleviate its related comorbidities.

*Ligusticum chuanxiong hort* (*Ligusticum wallichii*, L. chuanxiong) is one of the most commonly used Chinese herbs and has been harnessed by oriental medicine for the treatment of cardiovascular and cerebrovascular diseases (Yang et al., 2020). Previous studies showed that it could activate circulation, relieve pain, and be used for rheumatism and arthritis pain (Li et al., 2014; Liu et al., 2015; Hu et al., 2016). Tetramethylpyrazine (TMP) is an alkaloid extracted from Rhizoma Chuanxiong, and has been identified as the main active constituent from L. Chuanxiong (Jin et al., 2019). Several previous studies have also demonstrated that TMP potentially play a neuroprotective role in neurological disorders, such as brain ischemic or traumatic injury (Xiao et al., 2010; Tang et al., 2012; Kong et al., 2016). We recently also reported that there was an anti-epileptogenic effect of TMP on kindling models of epilepsy, but no protective effects on generalized seizures (GS) in kindled mice or MES- and PTZ-induced acute seizure (Jin et al., 2019). TMP also has a potential antidepressant-like effect in chronic unpredictable mild stress (CUMS) induced depressive mice (Fu et al., 2019). Despite these studies, the effects of TMP on the neuropsychiatric comorbidities in epilepsy and other neurological disease remain unclear.

On the other hand, arthritic pain is another typical neurological disease with serious psychiatric complications, and usually the initial symptoms in patients with clinical arthritis (Hunter et al., 2008). Arthritic pain often leads to avoidance, disability, sleep disorders, depression and anxiety (Cohen and Mao 2014; Rogal et al., 2015; Zale and Ditre 2015), which, in turn, lead to the persistence of the pain experience, thereby fueling the vicious circle of increasing depression and anxiety in clinic (Ohmichi et al., 2018). Most arthritic pain patients show poor response to conventional classic analgesics, such as non-steroidal anti-inflammatory analgesics and opioid analgesics (Lavin and Park 2011; Scherrer, Svrakic et al., 2014). Moreover, the higher morbidity and mortality risks associated with analgesic misuse are reported as a consequence of overdose treatments with the non-steroidal anti-inflammatory drug and/or paracetamol/codeine components (Cairns et al., 2016). As descripted above, the landscape opens the door to the exploration of novel therapeutic targets or specific drugs for arthritis pain, as well as the neuropsychiatric complications. In the present study, we investigated the effects of TMP on corneal kindling model of epilepsy, as well as on the initiation and maintenance of complete Freund’s adjuvant (CFA)-induced arthritic chronic pain. We also presented the data of the effects on their psychiatric comorbidities.

Continuous infusion of BDNF inhibited the development of behavioral seizures, which shed light on the role of BDNF in the progression of epileptogenesis (Xu et al., 2004; Zhang 2011). A large body of literature has also showed the major role for BDNF in pain pathophysiology, and its antidepressant effect by activating the ERK pathway (Zheng et al., 2017; Xia et al., 2020; Zhang et al., 2020). The BDNF-ERK might participate in the molecular mechanism of depression signaling pathway and be a potential target for antidepressants (Wang and Mao, 2019). However, whether the BDNF-ERK signaling is involved in the psychiatric comorbidities of neurological diseases is still unclear. The possible molecular mechanisms involved were further investigated, particularly focusing on early hippocampal BDNF-ERK signaling impairment of neurological models. The knowledge of the underlying mechanisms might allow better control the development of neurological disease, including epilepsy and arthritis pain, as well as the neuropsychiatric comorbidities.

## Methods

### Animals

C57/Bl6J male mice (8–10 weeks, 20 ± 2 g body weight) were purchased from the experimental animal center of Zhejiang Chinese Medical University [SCXK (Yu)-2005-3001, Zhejiang Province, P.R. China]. They were acclimatized for a week before testing in a standard light-and temperature-controlled animal facility at 22 ± 2°C, in 50 ± 10% relative humidity with a 12-h dark-light cycle with no more than five mice per cage, freely fed with plentiful food and water. The animals were treated and cared for in compliance with the Guide for the Care and Use of Laboratory Animals of the National Academy of Sciences (National Research Council (US) Institute for Laboratory Animal Research, 1996). The experimental protocol was approved by Animal Care and Use Committee of Zhejiang Chinese Medical University and our departmental ethics committee. Efforts were made to minimize the number and suffering of animals used.

### Reagents and Treatments

Complete Freund's adjuvant (CFA, cat# F5881) was purchased from Sigma United States. Before use, 5% CFA was prepared using physiological saline and stored in a refrigerator at 4°C. Tetramethylpyrazine (TMP, analytical standard, HPLC ≥ 98%, cat# B21436-20 mg) was purchased from Shanghai Yuanye Biotechnology Co., Ltd. The saline was purchased from Sinopharm Chemical Reagent Co., Ltd. In the experiment of epilepsy, TMP was administered from the initial day or from the 21st day of the Corneal (6 Hz) kindling, twice per day to the 35th day (50 mg/kg, i.p., D1-D35 vs. D21-D35), according to our previous work (Jin et al., 2019). In the CFA induced pain model, TMP was administered from the initial day or from the 14th day of the experiment, once per day to the 14th or 28th day (5, 10 or 25 mg/kg, i.p., D0-D14, D0-D28 vs. D14-D28), as indicated in the results section. The behavioral equipment was provided by the platform of the Experimental Animal Center of Zhejiang Chinese Medical University.

### Corneal (6 Hz) Kindling Model

The kindling model was conducted according to the protocol described by our previous reports (Jin et al., 2019). Groups of mice (*n* = 8) were stimulated twice daily through corneal electrodes connected to an ECT Unit 57800 stimulator (Ugo Basile) with a current intensity of 44 mA, 0.2 ms monopolar pulses at 6 Hz for 3 s duration, which initially induces only focal seizures. Seizure severity was assessed after each stimulate according to Racine's scale (Albertini et al., 2018). Fully kindled state was defined as 10 consecutive generalized seizures. For testing the effects of TMP on corneal kindling, saline and TMP (50 mg/kg, i.p., D1-D35 vs. D21-D35) were given twice per day, 15 min prior to each kindling stimulate.

### Complete Freund’s Adjuvant Injection

The model of arthritic inflammation was produced by performing four intra-articular injections of CFA (10 μl) at days 0, 7, 14 and 21 unilaterally into the right knee joint, as previously reported and validated (Torres-Guzman et al., 2014). Briefly, mice were anesthetized using isoflurane inhalation (3% for induction and 1.5% for maintains, v/v in air, respectively), followed by an intraarticular injection of CFA using a 28-gauge, 0.5-inch needle that was fitted with cannulation tubing, such that only 2–3 mm of the needle was allowed to puncture the joint. CFA was injected through the patellar ligament into the articular space using the femoral condyles as a guide.

### Measurement of Knee Thickness

The digital vernier caliper was purchased from East China Pharmaceutical Co., Ltd. The inflammation was quantified by measuring the knee thickness (in millimeter). The diameter of the knee joint was defined just below level of the patella and was measured in the anesthetized animal using a digital caliper on day 0 (before the CFA injection) and on day 7, 14, 21 and 28 after the initial injection of CFA.

### Arthritic Joint Pain Behavior Measurement

Measurements of touch allodynia (significant decrease in paw withdrawal threshold compared with baseline values) were carried out by using von Frey monofilaments as previously reported (Yu et al., 2019). Behavioral tests (*n* = 8–10 animals/group) were carried out on every 4 days after each CFA injection (4th, 11th, 18th and 25th days, PO) by blinded examiners. Animals were placed in a chamber with a mesh metal floor (20 × 30 cm), covered by an opaque plastic dome 10-cm high, and were always allowed to habituate for 1 h before any test. Tactile allodynia (i.e., a decreased threshold to paw withdrawal after probing with normally innocuous mechanical stimuli) was measured with a set of von Frey hairs (UGO basile, cat# 37450-275) with a bending force ranging from 0.02 to 4 g for the mice. Stimulation was applied to the plantar surface of the ipsilateral hind paw. Each hair was indented to the mid-plantar skin until it just bent. Clear paw withdrawal, shaking, or licking was considered as a nociception-like response. The filament of 0.7 g was used first. The strength of the next filament was decreased if the animal responded or increased if the animal did not respond. Withdrawal threshold was determined by sequentially increasing and decreasing the stimulus strength (the “up-and-down” method), and data were analyzed with Dixon’s nonparametric method, as described by Chaplan et al. (1994), and expressed as the mean withdrawal threshold.

As previously described (Torres-Guzman et al., 2014), the number of total vertical rearing was recorded during a 5-min observation period while the animals were in the open Plexiglas observation chamber. Total vertical rearing was defined as the number of times that the animals stood on both hind limbs while supporting their entire body weight.

### Mouse Brain Dissection and Collection

The day after the last behavioral test, all experimental animals were deeply anesthetized with sodium pentobarbital (50 mg/kg, i.p.), and the mPFC and hippocampus were isolated and collected. In brief, the skull was carefully opened to access whole brain, using curved forceps to take out the whole brain and making sure that no mechanical damage was done. The entire mPFC sections and hippocampus were dissected, put in a 1.5 ml Eppendorf tube, snap-frozen with liquid nitrogen, and then stored in a −80°C freezer.

### Western Blot Analysis

As previously reported (Lv et al., 2019), the collected mPFC sections and hippocampus were grinded into powders using liquid nitrogen, then transferred into 1.5 ml Eppendorf (EP) tubes and lysed in cold lysis buffer containing 1 mM phenyl methyl sulfonyl fluoride (PMSF). The samples were then vortexed at high speed for 15 s, incubated on ice for 15 min, and vortexed again at high speed for 15 s. After centrifugation (15,000 *g* for 15 min at 4°C), the total proteins obtained in the supernatant were quantified using BCA protein assay kit (Tiangen Biotech Co., Ltd., China), according to the manufacturer’s instructions. The proteins were mixed with 5x loading buffer and heated at 100°C for 3 min to denature. Western blot was then performed using 10% SDS-PAGE. Proteins were transferred to PVDF membranes (Merk, Germany) (83 mm × 75 mm). After 1 h blocking with 5% dried skim milk dissolved in PBST (0.05% Tween 20), the membranes were individually incubated with primary antibodies overnight at 4°C and then incubated with secondary antibody for 1 h. The data were analyzed via densitometry using Molecular Analyst software (Bio-Rad Laboratories, Hercules, California, United States) and quantitated levels were normalized to their respective blotting from GAPDH.

### Open-Field Test

As reported before (Kim et al., 2011), the open-field test (OFT) was performed in a 45-cm^2^ plastic box with 30-cm high walls. The OFT box was connected to a computer. Animals were placed at the center of the apparatus, and were observed for 5 min. The total distance traveled and the time spent in OFT were monitored and recorded by ANY-maze video-tracking software (Stoelting Co., IL, United States). The apparatus was cleaned with 75% alcohol between trials to avoid possible influence on locomotion.

### Elevated Plus Maze Test

The EPM consists of four elevated (30 cm) arms (30 cm long and 5 cm wide) with two opposing arms containing 30 cm high opaque walls. EPM testing occurred in a quiet testing room with ambient lighting at ∼50 lux (Fiorino et al., 2016). On day of testing, mice were allowed to acclimate to the testing room for 20 min. Each mouse was placed in a closed arm, facing the enter platform and cage mates started in the same closed arm. Each mouse was allowed 5 min to explore the EPM and then returned to its home cage. Between tests the EPM was cleaned thoroughly with 75% alcohol. EPM performance was recorded using an overhead video camera for later quantification. Open and closed arm entries were defined as the front two paws entering the arm, and open arm time began the moment the front paws entered the open arm and ended upon exit.

### Tail Suspension Test

The tail suspension test (TST) was conducted as initially described by Steru et al. (1985).The animals were individually suspended by the tail from a horizontal ring stand bar raised 30 cm above the ground, using adhesive tape placed 1 cm from the tip of the tail and positioned such that the base of the tail was aligned with the horizontal plane. The duration of the recording was 6 min. The latency and total immobility time during the last 4 min were measured for each animal in seconds. Immobility was considered in case of complete absence of all movements except for those required for respiration.

### Forced Swimming Test

The forced swimming test (FST) was performed according to the procedure of Doron et al. (2014) The test apparatus for mice consisted of a transparent cylindrical polypropylene tank (40 cm height × 20 cm diameter) containing 20 cm of water at 25 ± 1°C to prevent the animals from escaping. The water was changed between the tested animals. Mice were video recorded for 6 min. The latency and duration of immobility during the last 4 min were measured. Immobility corresponded to the total time spent floating motionless or making only movements to keep the head above the water surface.

### Statistical Analysis

The results were expressed as the mean ± sem and then analyzed by GraphPad Prism 8.0 (GraphPad Software, San Diego, CA, United States). Analyses of stages of kindling and pain behaviors were performed with repeated measures two-way ANOVA with Dunnett’s multiple comparisons. One-way analysis of variance (ANOVA) was used followed by Dunnett’s multiple comparisons for multiple groups comparisons. *p*-values < 0.05 were considered statistically significant.

## Results

### Tetramethylpyrazine Administered From the Initial Day, But Not in the Late Period, of Corneal Kindling Retarded the Progression of Epilepsy

**FIGURE 1:**
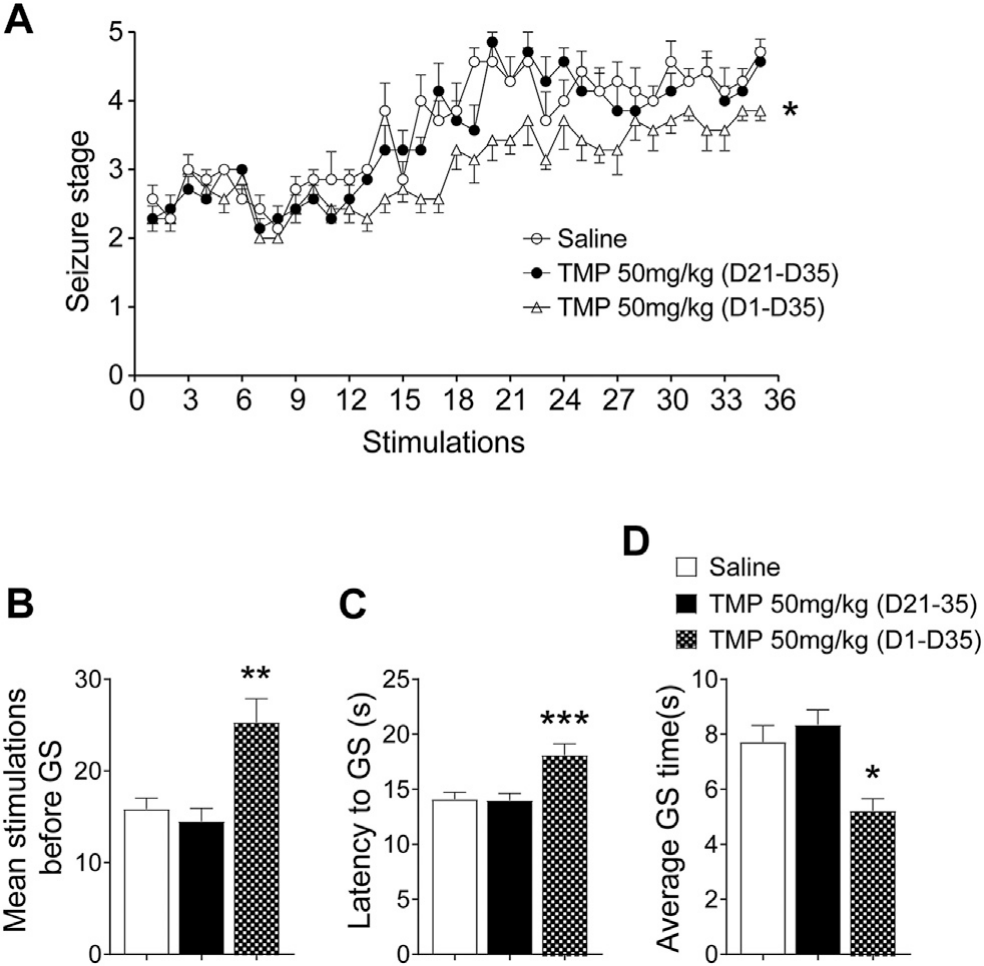
Time dependent effects of TMP on 6 Hz corneal kindling model. **(A)** Progression in seizure severity during kindling acquisition with TMP (50 mg/kg, i.p. D21-D35 vs. D1-D35 vs. Saline) administration. Statistical comparison between seizure scores of two groups was performed with a two-way repeated-measures ANOVA followed by a post hoc *t* test (*p < 0.05). **(B)** Number of stimulations required to reach the fully kindled state during the acquisition phase of the 6 Hz corneal kindling model. **(C)** Average latency and **(D)** average time of generalized seizures, during the acquisition phase of the 6 Hz corneal kindling model. Analyses of stages of kindling were performed with repeated measures two-way ANOVA with Dunnett’s multiple comparisons. One-way analysis of variance (ANOVA) was used followed by Dunnett’s multiple comparisons in **(B–D)**. (*p < 0.05, **p < 0.01 and ***p < 0.001 vs. Saline).

First, we observed the effects of 50 mg/kg TMP (dose reported effective on the epileptogenesis in our previous work) on the progression of epileptogenesis, as well as on the seizure severity in a 6 Hz corneal rapid kindling model, either administrated intraperitoneally from the initial day (50 mg/kg, i.p., D1-D35), or from the 21st day (50 mg/kg, i.p., D21-D35) when the mice were fully kindled as reported by our previous work (Jin et al., 2019) (Figures 1A–D). Two-way repeated-measures ANOVA analysis revealed significant effects of time [F (10.34, 186.2) = 24.87, *p* < 0.01], treatment [F (2, 18) = 39.32, *p* < 0.01], and interaction between time and treatment [F (68, 612) = 1.348, *p* = 0.0389]. Compared to saline group, the number of stimulates before reaching the fully kindled state was increased by 50 mg/kg TMP (i.p., D1-D35) administration during kindling acquisition [TMP (D1-D35) vs. Saline, 25.3 ± 2.6, *n* = 6 vs. 15.8 ± 1.2, *n* = 7, *p* < 0.01, Figure 1B]. One way ANOVA showed significant effects of treatment (time windows) on the number of stimulates between groups [F (2, 16) = 9.598, *p* < 0.01]. Moreover, one way ANOVA showed significant effects of treatment (time windows) on average latency [F (2, 268) = 6.169, *p* < 0.01] and the lasting time [F (2, 268) = 7.968, *p* < 0.01] of GS between groups (Figures 1C,D). Compared to the saline treated group, the average latency [TMP (D1-D35) vs. Saline, 18.1 ± 1.0, vs. 14.1 ± 0.6, *p* < 0.01, Figure 1C] and the lasting time [TMP (D1-D35) vs. Saline, 5.2 ± 0.4 vs. 7.7 ± 0.6, *p* < 0.01, Figure 1D] of generalized seizures (GS) induced by 6 Hz corneal kindling were reduced by TMP (D1-D35) administration. However chronic 50 mg/kg TMP (D21-D35) administration on the late phase showed no effect on the epileptogenesis and the GS in the 6 Hz corneal kindling in mice, as shown in Figures 1A–D.

### Tetramethylpyrazine Administered From the Initial Day of Corneal Kindling Alleviated the Anxiety and Depressive Behaviors, But Late Period Administration Did Not

**FIGURE 2:**
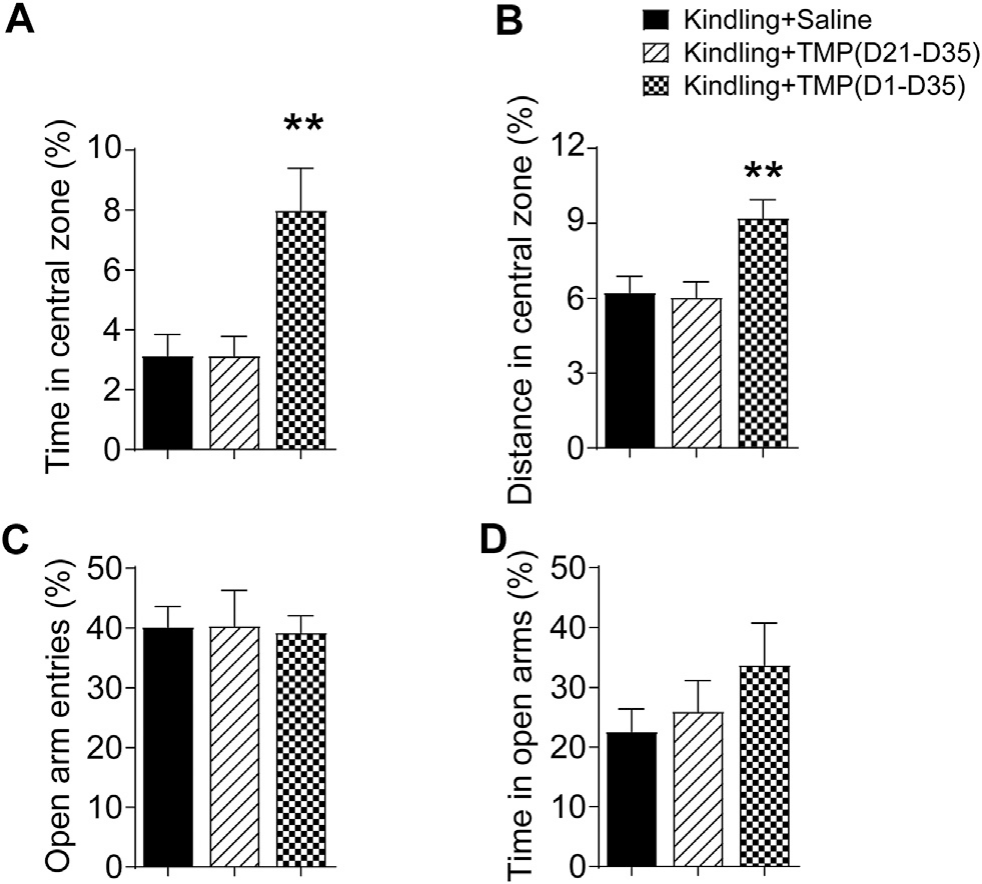
Time dependent effects of TMP on anxiety behaviors in late corneal kindling. **(A, B)** Effects on time **(A)** and distance percentage **(B)** in central zone of open field test with TMP (50 mg/kg, i.p. D21-D35 vs. D1-D35) administration. **(C, D)** Effects on entries **(C)** and time percentage **(D)** in open arms of EPM test with TMP (50 mg/kg, i.p. D21-D35 vs. D1-D35 vs. Saline) administration. Statistical analysis was performed by one way ANOVA, followed by post Dunnett’s multiple comparisons (**p < 0.01, vs. Kindling + Saline).

We next determined the effects of 50 mg/kg TMP chronic administration with different treatment periods (50 mg/kg, i.p., D1-D35 vs. D21-D35) on the depressive and anxiety-like behaviors, which were reported as popular psychiatric comorbidities in epilepsy (Gaitatzis et al., 2004). Open-field test (OFT) and elevated plus maze (EPM) are two common models used to test the anxiety-like behaviors. Mice always tend to stay in darker places, avoiding strongly illuminated places; but at the same time, they prefer to explore novel areas. It is also considered that the entering and exploring to the central zone of an open field is inversely correlated to anxiety levels of the mice. Thus, as shown in Figures 2A,B, one way ANOVA showed significant effects of treatment (time windows) on the percentage of time spent [F (2, 18) = 8.061, *p* < 0.01] and traveling distance [F (2, 18) = 7.116, *p* < 0.01] in the central zone. TMP administrated from the initial day (D1-D35) of the 6 Hz corneal kindling increased the ratio of exploring time (D1-D35 vs. saline, 8.0%, *n* = 7 vs. 3.1%, *n* = 7, *p* < 0.01) and travelling distance [D1-D35 vs. saline, (9.2 ± 0.7) % vs. (6.2 ± 0.6) %, *p* < 0.05] in the central zone of open field test. However, TMP late phase administration (D21-D35) that did not affect the GS in kindling also showed no effect on the anxiety like behaviors in the OPT (Figures 2A,B). Our results showed no significant difference in open arm entries [F (2, 18) = 0.01936, *p* > 0.05] and time [F (2, 18) = 1.090, *p* > 0.05], as well as in close arm entries [F (2, 18) = 0.4249, *p* > 0.05], between three groups in the EPM experiments (Figures 2C,D; Supplementary Figure S1A).

**FIGURE 3:**
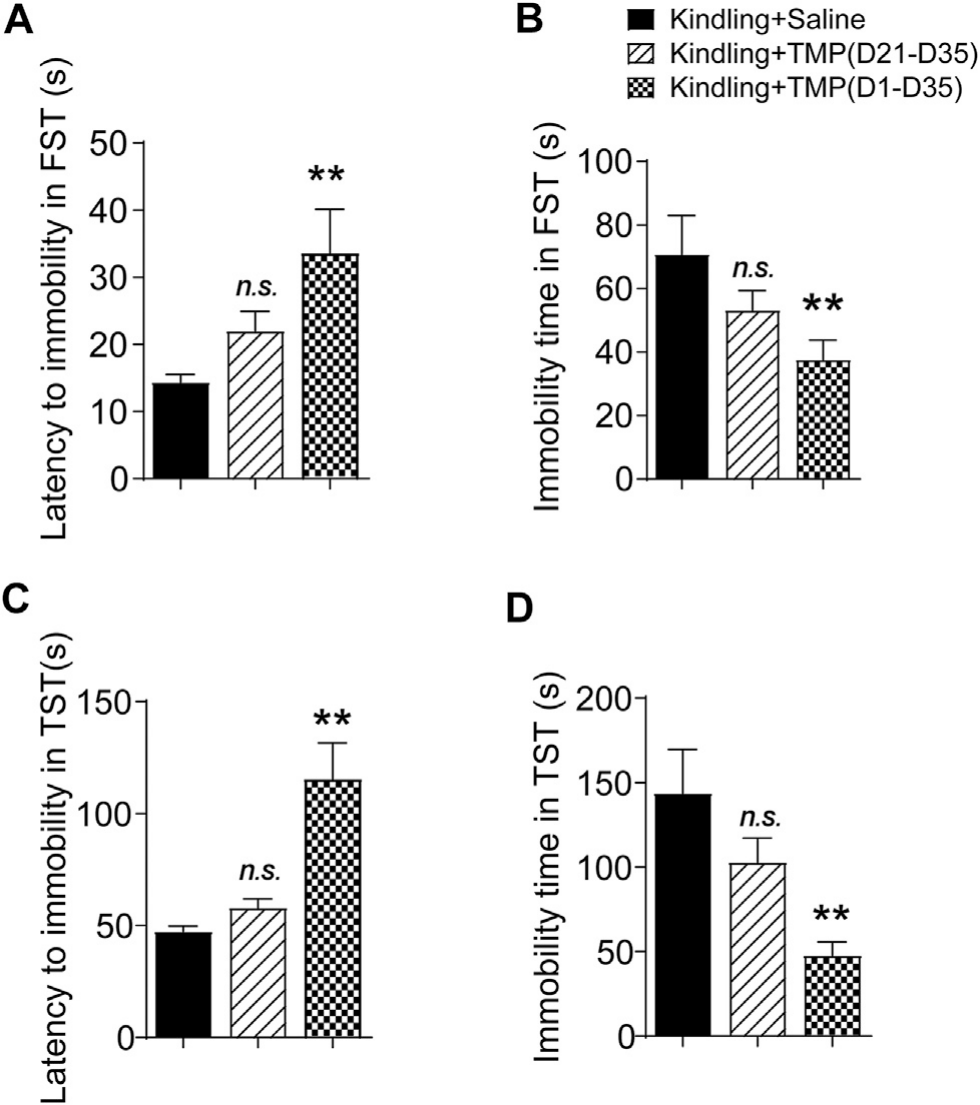
Time dependent effects of TMP on depressive behaviors in late corneal kindling. **(A, B)** Effects on latency to immobility **(A)** and total immobility time **(B)** in FST test with TMP (50 mg/kg, i.p. D21-D35 vs. D1-D35 vs. saline) administration. **(C, D)** Effects on latency to immobility **(C)** and total immobility time **(D)** in TST test with TMP (50 mg/kg, i.p. D21-D35 vs. D1-D35 vs. saline) administration. Statistical analysis was performed by one way ANOVA, followed by Dunnett’s multiple comparisons (**p < 0.01, vs. Kindling + Saline).

The forced swimming test (FST) and tail suspension test (TST) are two animal tests commonly used to assess depressive like behaviors. Both are evaluated by the latency time recorded before mice giving up attempts to escape and become immobile in an aversive situation, as well as the total immobile time during recording. Similarly, we observed the effects of 50 mg/kg TMP (i.p., D1-D35 or i.p. D21-D35) on the depressive behaviors in the FST and TST tests, one way ANOVA showed significant effects of treatment (time windows) on latency [F (2, 17) = 4.745, *p* < 0.05] and total immobility time [F (2, 18) = 3.717, *p* < 0.05] in FST.TMP administration (D1-D35) increased the latency (D1-D35 vs. saline, 33.6 ± 6.6, *n* = 6 vs. 14.3 ± 1.2, *n* = 7, *p* < 0.05) and total immobility time (D1-D35 vs. saline, 37.6 ± 6.2, *n* = 7 vs. 70.9 ± 12.1, *n* = 7, *p* < 0.05)in FST, compared to saline group (Figures 3A,B). On the contrary, TMP late period administration (D21-D35) had no effects on the mice behaviors in FST. Furthermore, one way ANOVA showed significant effects of treatment (time windows) on latency [F (2, 18) = 13.95, *p* < 0.01] and total immobility time [F (2, 18) = 7.311, *p* < 0.01] in TST, as shown in Figures 3C,D. TMP late period administration also increased the latency to immobility (D1-D35 vs. saline, 115.3 ± 16.3, *n* = 7 vs. 47.3 ± 2.5, *n* = 7, *p* < 0.01), and decreased the total immobility time (D1-D35 vs. saline, 47.7 ± 8.0, *n* = 7 vs. 143.6 ± 26.1, *n* = 7, *p* < 0.01) in TST experiment (Figures 3C,D). However, compared to the saline group, TMP late period administration (50 mg/kg, i.p., D21-D35) did not show any effects on the depressive behaviors in the TST (Figures 3C,D).

### Tetramethylpyrazine Administered From the Initial Day of Corneal Kindling Increased the Hippocampal BDNF/ERK Expression

**FIGURE 4:**
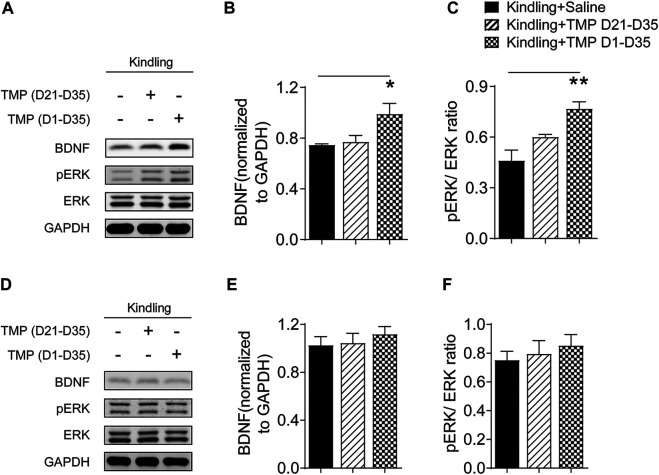
Time dependent effects of TMP on hippocampal BDNF/ERK expression in late corneal kindling. **(A–C)** Levels of hippocampal BDNF **(B)** and pERK/ERK ratio **(C)** with TMP (50 mg/kg, i.p. D21-D35 vs. D1-D35 vs. saline) administration in late corneal kindling. **(D–F)** Levels of BDNF **(E)** and pERK/ERK ratio **(F)** in mPFC with TMP (50 mg/kg, i.p. D21-D35 vs. D1-D35 vs. saline) administration. Statistical analysis was performed by one way ANOVA, followed by Dunnett’s multiple comparisons (*p < 0.05, **p < 0.01, vs. Kindling + Saline).

Impaired BDNF/ERK signaling has a crucial role in the development of epileptogenesis (Xu et al., 2004), as well as the neuropsychiatric comorbidities (Wang and Mao 2019), so we further determined if TMP (50 mg/kg, i.p., D1-D35) improved anxiety and depressive like behaviors by increasing hippocampal BDNF/ERK signaling by Western blotting. One way ANOVA showed significant effects of treatment (time windows) on BDNF [F (2, 9) = 5.564, *p* < 0.05] and pERK/ERK expression [F (2, 9) = 12.07, *p* < 0.01] in hippocampus. As shown in Figure 4, compared to saline group, TMP administration (D1-D35) increased the protein expression of BDNF [TMP (D1-D35) vs. Saline, 0.99 ± 0.08, *n* = 4 vs. 0.75 *±* 0.01, *n* = 4, *p* < 0.01] and phosphorylated ERK [TMP (D1-D35) vs. Saline, 0.77 ± 0.04 vs. 0.46 ± 0.06, *n* = 4, *p* < 0.01] in hippocampus (Figures 4A–C). In contrast, TMP administration in the late phase (D21-D35) had no effect on the expression of BDNF and phosphorylated ERK (Figures 4A–C). There was no difference in the expression of BDNF and phosphorylated ERK in mPFC between three groups in our experiment (Figures 4D–F).

### Tetramethylpyrazine Administered in the Late Period of Complete Freund’s Adjuvant Induced Arthritis Pain Attenuated Knee Edema and Pain-Related Behaviors

We have already shown that TMP administration (D1-D35) retarded the epileptogenesis, as well as the anxiety and depressive comorbidities, whereas the late phase administration of TMP (D21-D35) had no effect on both of the seizures and its neuropsychiatric comorbidities. Then, we further evaluated the effects of TMP on the development of inflammatory pain, which is another neurological disorder with clearly studied anxiety and depressive comorbidities.

**FIGURE 5:**
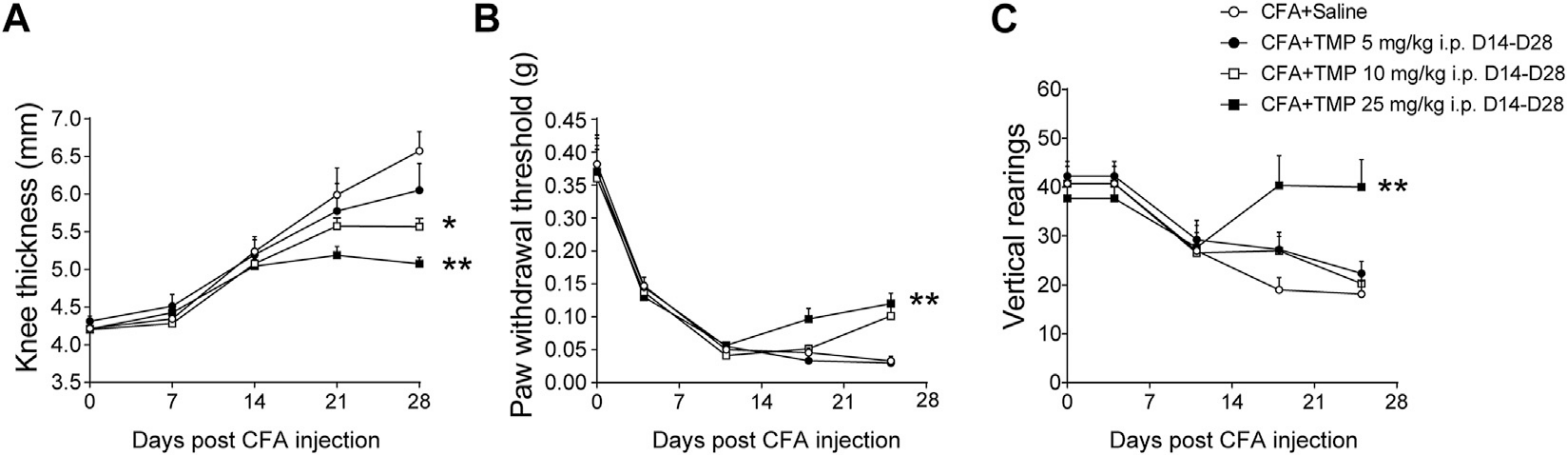
Dose dependent effects of TMP on maintenance of CFA induced inflammatory pain. **(A)** The effect of TMP on knee thickness. **(B)** The mechanical pain threshold between groups. **(C)** The vertical rearings with TMP administration between groups. Statistical comparison was performed with repeated measures two-way ANOVA with post Dunnett’s multiple comparisons. (*p < 0.05, **p < 0.01, vs. CFA+Saline).

First, we determined the dose dependent effects of TMP on CFA induced arthritis pain. As shown in Figure 5A, two-way repeated-measures ANOVA analysis revealed significant effects of time [F (1.989, 57.68) = 30.71, *p* < 0.01], treatment [F (3, 29) = 3.566, *p* < 0.05], and interaction between time and treatment [F (6, 58) = 5.079, *p* < 0.01] on the knee thickness of mice increased after CFA injection. TMP (10 and 25 mg/kg i.p., D14-D28) decreased the knee thickness in the development of chronic pain modeling (Figure 5A), but 5 mg/kg TMP administration had no effects. Moreover, two-way repeated-measures ANOVA analysis revealed significant effects of time [F (1.754, 45.60) = 3.592, *p* = 0.041], treatment [F (3, 26) = 10.31, *p* < 0.01], and interaction between time and treatment [F (6, 52) = 5.244, *p* < 0.01] on the paw withdraw thresholds in von Frey test. Mice from TMP (25 mg/kg, i.p., D14-D28) administrated groups showed significantly higher paw withdraw thresholds, compared to the mice from saline group (*p* < 0.01, vs. saline, Figure 5B). Vertical rearing (use of both hind limbs for supporting the entire body weight) is used to determine behavioral changes in relation to nociception. Thus, we further tested the effects of administration of TMP with different dosages on the vertical rearing behaviors (Figure 5C). Furthermore, two-way repeated-measures ANOVA analysis revealed significant effects of time [F (1.707, 40.98) = 1.246, *p* > 0.05], treatment [F (3, 24) = 5.242, *p* < 0.01], and interaction between time and treatment [F (6, 48) = 3.133, *p* < 0.05] on the vertical rearing. Compared to saline group, only the mice from TMP (25 mg/kg, i.p., D14-D28) late phase administration group showed more vertical rearing in 5 min test periods (Figure 5C).

**FIGURE 6:**
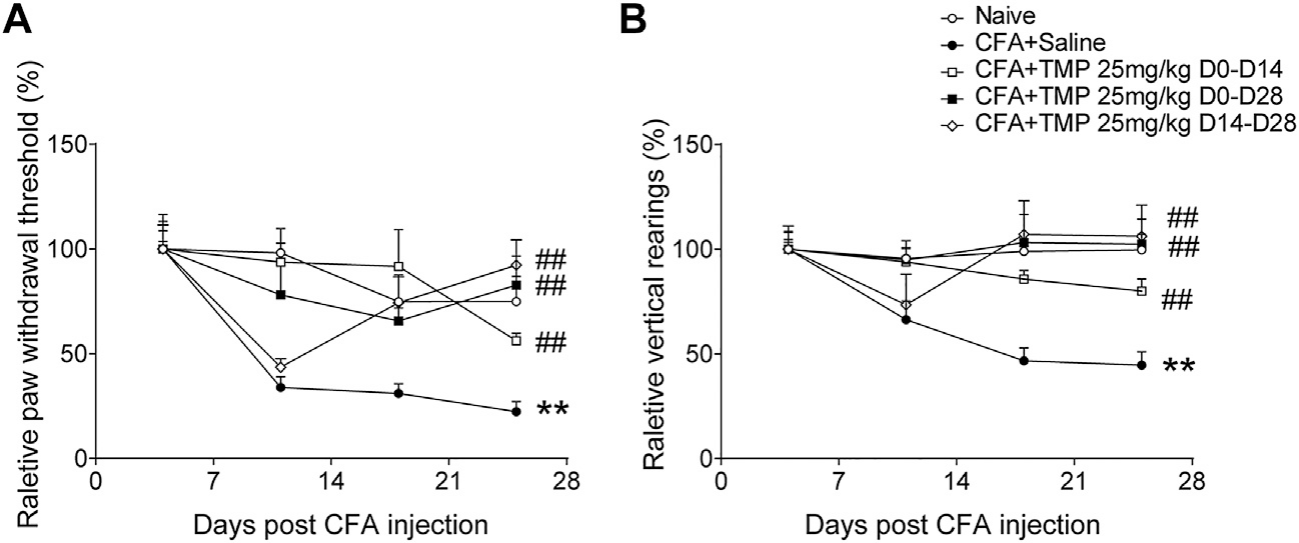
The analgesic effects of TMP on the initiation and maintenance of CFA induced pain. **(A)** The relative paw mechanical pain threshold and **(B)** vertical rearings with different TMP administration periods. Statistical comparison was performed with repeated measures two-way ANOVA with post Dunnett’s multiple comparisons (##*p* < 0.01, vs. CFA+Saline; **p < 0.01, vs. Naïve).

In addition, we compared the effects of 25 mg/kg TMP on the arthritis pain behaviors when it was administered with different periods (D0-D28, D0-D14 and D14-D28) after CFA injection. Two-way repeated-measures ANOVA analysis revealed significant effects of time [F (1.989, 57.68) = 30.71, *p* < 0.01], treatment (time windows) [F (3, 29) = 3.566, *p* < 0.05], and interaction between time and treatment (time windows) [F (6, 58) = 5.079, *p* < 0.01] on paw withdraw thresholds (Figure 6A). Moreover, Two-way repeated-measures ANOVA analysis also revealed significant effects of time [F (2.161, 64.83) = 5.024, *p* < 0.01], treatment (time windows) [F (4, 30) = 5.295, *p* < 0.01], and interaction between time and treatment (time windows) [F (12, 90) = 3.997, *p* < 0.01] on vertical rearing (Figure 6B). As shown in Figure 6, compared to saline group, all the three treatments of TMP increased paw withdraw thresholds and vertical rearings in the development of chronic pain modeling (Figures 6A,B).

### Tetramethylpyrazine Administered From the Initial Day of Complete Freund’s Adjuvant Injection Alleviated Anxiety and Depressive-like Behaviors, but Late Administration did Not

**FIGURE 7:**
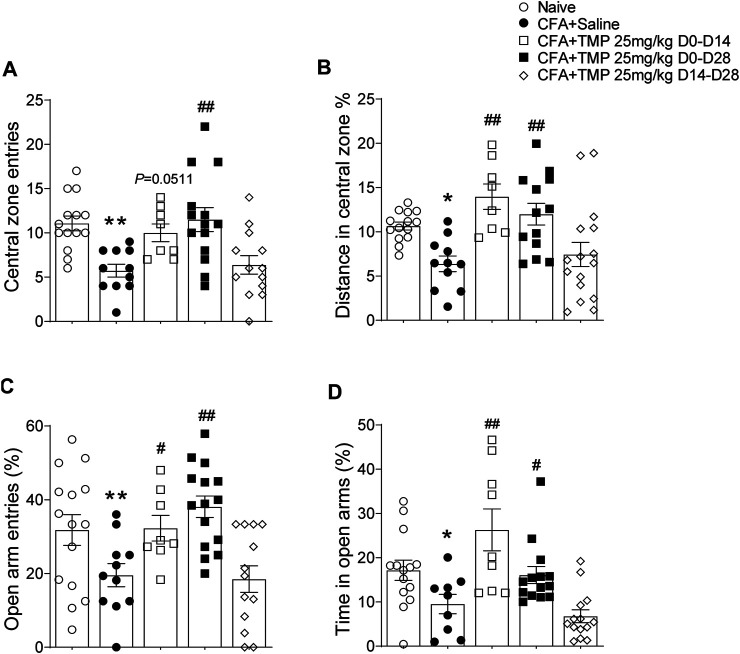
Time dependent effects of TMP on anxiety behaviors in CFA induced pain. **(A, B)** Effects on entries **(A)** and distance percentage **(B)** in central zone of open field test with different TMP administration periods. **(C, D)** Effects on entries **(C)** and time percentage **(D)** in open arms of EPM test with TMP with different TMP administration periods as indicated (25 mg/kg, i.p. D0-D14, D0-D28 and D14-D28 vs. saline). Statistical analysis was performed by one way ANOVA, followed by post Dunnett’s multiple comparisons (*p < 0.05, **p < 0.01, vs. Naïve; #*p* < 0.05, ##*p* < 0.01, vs. CFA+Saline).

Since the TMP administration has no time window dependent effects on the arthritis pain, we further investigated the effects of TMP, employed different administration periods (D0-D14, D0-D28, D14-D28 vs. saline), on anxiety and depressive-like behaviors in late inflammatory pain. One way ANOVA showed significant effects of treatment (time windows) on the entrance [F (4, 55) = 6.698, *p* < 0.01] and traveling distance percentage [F (4, 57) = 6.634, *p* < 0.01] in the central zone (Figures 7A,B). Compared to saline group, the mice from TMP (D0-D14 and D0-D28) treatment groups showed more central zone entrances (D0-D14 vs. saline, 10.0 ± 1.0, *n* = 8 vs. 5.7 ± 0.7, *n* = 11, *p* = 0.0511; D0-D28 vs. saline, 11.5 ± 1.4, *n* = 14 vs. 5.7 ± 0.7, *p* < 0.01, Figure 7A) and higher ratio of central zone distance (D0-D14 vs. saline, 14.0% ± 1.4%, *n* = 8 vs. 6.4% ± 0.9%, *n* = 11, *p* < 0.01; D0-D28 vs. saline, 14.0% ± 1.4%, *n* = 13 vs. 6.4% ± 0.9%, *n* = 11, *p* < 0.01, Figure 7B). Furthermore, one way ANOVA showed significant effects of treatment (time windows) on the percentage of open arms entrance [F (4, 57) = 5.959, *p* < 0.01] and time spent [F (4, 55) = 8.506, *p* < 0.01] in EPM. The mice from TMP (D0-D14 and D0-D28) treatment group also showed high ratio of entries (D0-D14 vs. saline, 32.3% ± 3.5% *n* = 8 vs. 19.6% ± 3.1%, *n* = 11, *p* < 0.05; D0-D28 vs. saline, 38.1% ± 2.9%, *n* = 15 vs. 19.6% ± 3.1%, *n* = 11, *p* < 0.01) and time spent (D0-D14 vs. saline, 16.1% ± 1.9%, *n* = 9 vs. 9.5% ± 2.2%, *n* = 9, *p* < 0.05; D0-D28 vs. saline, 16.1% ± 1.9%, *n* = 14 vs. 9.5% ± 2.2%, *n* = 9, *p* < 0.01) in open arms of EPM (Figures 7C,D). However, TMP treatment started from the 14th day after the initial injection of CFA (D14-D28) had no effect on the anxiety-like behaviors in both tests (Figure 7). We did not find any difference in close arm entries [F (4, 57) = 0.1619, *p* > 0.05] between groups in the EPM experiments (Supplementary Figure S1B).

**FIGURE 8:**
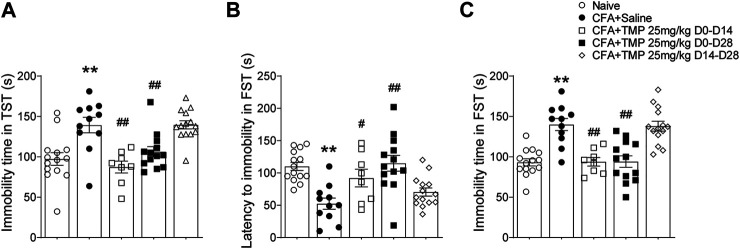
Time dependent effects of TMP on depressive behaviors in CFA induced pain. **(A)** Effects on total immobility time in TST test with different TMP administration periods (25 mg/kg, i.p. D0-D14, D0-D28 and D14-D28 vs. saline). **(B, C)** Effects on latency to immobility **(B)** and total immobility time **(C)** in FST test with different TMP administration periods as indicated. Statistical analysis was performed by one way ANOVA, followed by Dunnett’s multiple comparisons (**p < 0.01, vs. Naïve; #*p* < 0.05, ##*p* < 0.01, vs. CFA+Saline).

We further examined the effects of TMP on depressive like behavior using the forced swim test and tail suspension test (Figure 8). Similarly, one way ANOVA showed significant effects of treatment (time windows) on total immobility time [F (4, 55) = 10.32, *p* < 0.01 for TST; F (4, 55) = 15.89, *p* < 0.01 for FST] in both TST and FST, as well as on latency of immobility [F (4, 55) = 8.418, *p* < 0.01] in FST test. The mice from TMP (D0-D14 and D0-D28) groups had less total immobility time both in TST (D0-D14 vs. saline, 87.3 ± 7.3 s, *n* = 8 vs. 139.3 ± 9.6 s, *n* = 11, *p* < 0.01; D0-D28 vs. saline, 106.3 ± 6.3 s, *n* = 13 vs. 139.3 ± 9.6 s, *n* = 11, *p* < 0.01) and FST (D0-D14 vs. saline, 93.9 ± 5.3 s, *n* = 8 vs. 139.9 ± 7.5 s, *n* = 11, *p* < 0.01; D0-D28 vs saline, 94.1 ± 7.0 s, n = 13 vs. 139.9 ± 7.5 s, *n* = 11, *p* < 0.01) tests (Figures 8A,C), as well as longer latency of immobility (D0-D14 vs. saline, 92.1 ± 13.7 s vs. 52.5 ± 8.6 s, *p* < 0.05; D0-D28 vs. saline, 115.0 ± 12.2 s vs. 52.5 ± 8.6 s, *p* < 0.01) in FST test (Figure 8B). On the contrary, TMP treatment started from the 14th day after the initial injection of CFA (D14-D28), when the inflammatory pain has been established, had no effect on the depressive like behaviors (Figure 8).

### Tetramethylpyrazine Administered From the First Day of Initial Complete Freund’s Adjuvant Injection Increased the Hippocampal BDNF/ERK Expression, But Late Phase Administration Did Not

**FIGURE 9:**
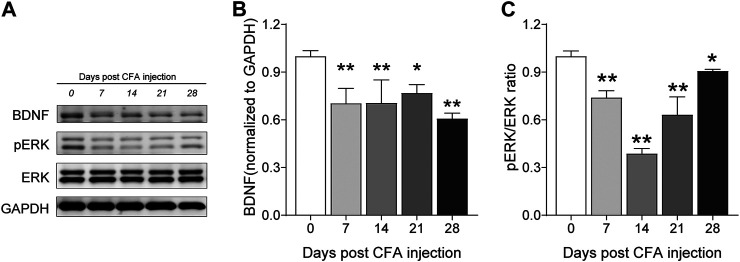
Time course of hippocampal BDNF-ERK expression after CFA injection. **(A–C)** Levels of hippocampal BDNF **(B)** and pERK/ERK ratio **(C)** by western blot testing after CFA injection. Statistical analysis was performed by one way ANOVA, followed by Dunnett’s multiple comparisons (*p < 0.05, **p < 0.01, vs. Day 0).

**FIGURE 10:**
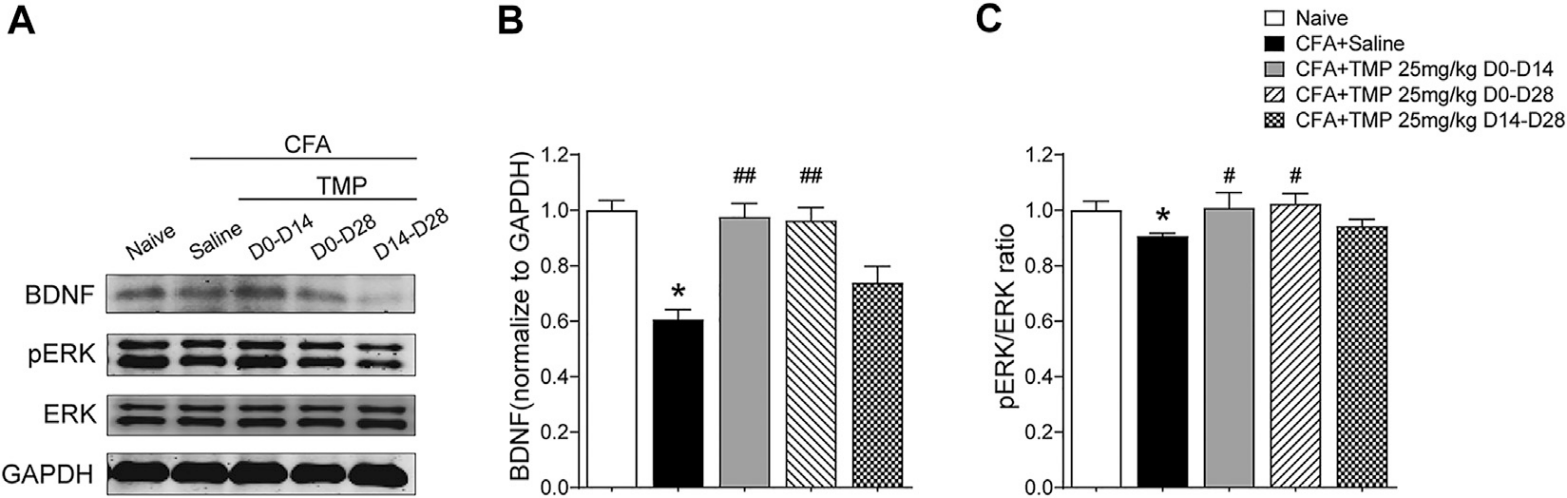
Time dependent effects of TMP on hippocampal BDNF/ERK expression in CFA induced inflammatory pain. **(A–C)** Levels of hippocampal BDNF **(B)** and pERK/ERK ratio **(C)** with different TMP administration periods (25 mg/kg, i.p. D0–D14, D0–D28 and D14–D28 vs. saline). Statistical analysis was performed by one way ANOVA, followed by Dunnett’s multiple comparisons (*p < 0.05, vs. Naïve; #*p* < 0.05, ##*p* < 0.01, vs. CFA+Saline).

Then, we investigated the time course of hippocampus BDNF-ERK signaling variation after CFA injection. One way ANOVA showed significant effects of CFA injection on BDNF expression [F (4, 22) = 10.56, *p* < 0.01]. We found that BDNF decreased on 7th, 14th, 21st and 28th day after CFA injection (Day 7, 14, 21, 28 vs. Day 0, 0.70 ± 0.09, 0.71 ± 0.15, 0.77 ± 0.05, 0.61 ± 0.03 vs. 1.0 ± 0.00; *p* < 0.01, *p* < 0.01, *p* < 0.05, *p* < 0.01; Figures 9A,B). In addition, one way ANOVA also showed significant effects of CFA injection on pERK expression [F (4, 22) = 25.08, *p* < 0.01]. pERK/ERK decreased on 7th, 14th, 21st and 28th day after CFA injection too, although the level on 14th day seems to be the lowest one (Day 7, 14, 21, 28 vs. Day 0, 0.74 ± 0.04, 0.39 ± 0.03, 0.63 ± 0.11, 0.89 ± 0.02 vs. 1.0 ± 0.03, *p* < 0.05, *p* < 0.05, *p* < 0.01, *p* < 0.05; Figure 9C). Finally, we determined the effects of TMP administration with different periods on the BDNF/ERK expression in mice with inflammatory pain. One way ANOVA showed significant effects of treatment (time windows) on BDNF expression [F (4, 30) = 17.97, *p* < 0.01] and pERK/ERK [F (4, 30) = 2.78, *p* < 0.05] in hippocampus (Figures 10A–C). It was shown that, compared to saline group, TMP administration from first day of initial CFA injection (D0-D14 and D0-D28) increased the protein expression of BDNF (D0-D14, 0.99 ± 0.02, *n* = 6; D0-D28, 0.96 ± 0.05, *n* = 6; vs. Saline 0.61 ± 0.03, *n* = 6; *p* < 0.01; *p* < 0.01; Figure 10A) and phosphorylated ERK (D0-D14, 1.02 ± 0.03, *n* = 6; D0-D28, 1.02 ± 0.04, *n* = 6; vs. Saline 0.89 ± 0.01, *n* = 6; *p* < 0.05; *p* < 0.05; Figure 10C) in hippocampus, whereas TMP administration (D14-D28) had no effect on it (Figures 10A–C). We did not see any difference in the expression of BDNF and phosphorylated ERK in mPFC between the three groups (data not shown).

## Discussion

Anxiety and depressive-like behaviors are recognized as common co-morbidities in many neurological diseases. Moreover, there is mounting evidence showing a bi-directional association that patients with psychiatric disorders history are more likely to have a higher risk of neurological diseases. In the present study, we found that: 1) TMP administered from the initial day (D1-D35 in kindling model, D0-D14 and D0-D28 in CFA model) of modeling retarded both the developments of 6 Hz corneal rapid kindling epileptogenesis and CFA induced inflammatory pain. 2) Late periods administration of TMP (D21-D35 in kindling, D14-D28 in CFA model) showed no effect on the epileptogenesis and the GS of kindling, but maintains alleviation in CFA induced inflammatory pain. 3) TMP treatments from the initial day of modeling (D1-D35 in kindling model, D0-D14 and D0-D28 in CFA model) alleviated the co-morbid depressive and anxiety-like behaviors in both models; however, late periods treatments did not, either in kindling or the CFA induced inflammatory pain. 4) TMP administered from the initial day of modeling increased the hippocampal BDNF/ERK expression, whereas late period administration showed no effects.

We previously reported that TMP suppressed the activity of calcium channels, but had no effect on sodium channels in hippocampal neurons (Jin et al., 2019). TMP also selectively suppressed the JNK signal pathway to inhibit the activation of astrocytes and then attenuated neuropathic pain via downregulation of TAK1 phosphorylation (Jiang et al., 2017). Consistent with our previous study in hippocampal kindling, we found that chronic TMP (50 mg/kg, i.p., D1-D35) administration had an anti-epileptogenic effect against corneal electrical kindling in mice, but did not promote anti-convulsive effects against GS in kindled mice (50 mg/kg, i.p., D21-D35). Nevertheless, different from its effect on the epileptogenesis and seizures, we further found that TMP administration prevented not only the development (25 mg/kg, i.p., D0-D28), but also the maintenance of mechanical hypersensitivity induced by CFA injection (25 mg/kg, i.p., D14-D28). Intra-articular injection of CFA could induce acute mechanical hypersensitivity that is independent of immune system in the early phase, but by the direct activation of nociceptors instead (Chiu et al., 2013). By acting on ATP receptor-ion channel complex (P2X receptors), TMP inhibited primary afferent transmission in DRG neurons (Liang et al., 2005; Gao et al., 2008). Thus, we inferred that the analgesic effect of TMP on the maintenance of chronic pain might be mediated by its modulation of the peripheral nociceptors or the activities of primary sensory neurons. Further experiments were needed to study this. But at least, our results firstly shed light on the importance of clinical application of TMP for arthritic chronic pain.

In animal models, the affective consequences of neurological disease (such as chronic pain) are reported time-dependent, usually requiring 2–4 weeks for anxiety-like and 6–8 weeks for depressive like behaviors to fully develop (Roeska et al., 2009; Yalcin et al., 2011). This raised another difficult but intriguing question that whether neurological disease pathological progress, such as epileptogenesis or the development of chronic pain, is etiologically associated with psychiatric comorbidity. It is worthy but difficult to assess whether the psychiatric disorders were a consequence of the neurological disease or pre/co-existing and even favored the development of neurological disease (Blackburn-Munro and Blackburn-Munro, 2001). In our present study, we found that TMP administered from the initial day of modeling retarded both 6 Hz corneal rapid kindling epileptogenesis and the development of CFA induced chronic pain, as well as their psychiatric co-morbidities. On the contrary, late period administration of TMP did not show any effects on both of the GS and the following co-morbid depressive and anxiety-like behaviors. Moreover, late administration of TMP even though resulted in the abrogation of inflammatory pain behaviors, but had no effects on the psychiatric comorbidity of chronic arthritic pain, either. Pharmacological or surgical management of the seizure disorder can have either a negative or a positive impact on psychiatric and neurological comorbidities (Kanner 2016). Of particular interest, although the various effects of TMP may be model dependent, we prefer to interpret our present data as indicating an early period of interference might be crucial for the alleviation of neurological diseases, and the relative psychiatric comorbidity as well. We further compared the effects on psychiatric comorbidity with different TMP administration periods in CFA induced chronic pain, and the results showed that both TMP (D0-D14 and D0-D28) treatments that started from the initial day had significant anti-psychiatric comorbidity effects (Figures 7C,D). A variety of things can lead to epilepsy and chronic pain, which may include traumatic brain injury, very high fever, stroke, and infectious diseases, etc. (Wilson et al., 2017; Thijs et al., 2019; Cohen et al., 2021; Offringa et al., 2021) Parasitic infection of the brain are also common causes of epilepsy and usually be controlled with medication, but it causes seizures at a later time (Monk et al., 2021). As a result, although it may not be applicable for the patients with neurological diseases that pathology fully developed, our present results at least indicated that the early period administration of TMP on neurological diseases modeling progression might be of great importance for controlling psychiatric comorbidities.

Clinical imaging demonstrates the morphological changes and dysfunction of brain structures accompanying neurological diseases, some of which are also concerned for their implications in psychiatric disorders, such as those observed the prefrontal cortex and the hippocampus (Mutso et al., 2012; Jackson 2013; Labate et al., 2020). It is also reported that chronic monoarthritis in rats induced anxious-depressive behaviors which coincided with increased extracellular signal regulated kinases (ERK) 1/2 phosphorylation in the spinal cord, hippocampus and the prefrontal cortex (Borges et al., 2014). Rats with chronic constrictive injury (CCI) of the sciatic nerve also showed decreased expression levels of ERK1 and p-ERK1/2 proteins in the hippocampus tissues (Wang et al., 2015). In addition, a variety of epilepsy models have shown that seizures induce transient increase of BDNF in neurons, in spite of the effects of BDNF in the development of behavioral seizures with different kindling paradigms are controversial, according to the previous findings (Reibel et al., 2000a; Reibel et al., 2000b; Scharfman et al., 2002). Overexpression of BDNF in ventral hippocampal CA1-infralimbic cortex reverses spontaneous pain, and accelerates recovery from inflammatory pain (Ma et al., 2019). Several studies have shown that BDNF could inhibit the development of kindling-induced seizures (Xu et al., 2004; McNamara and Scharfman 2012). However, the association between central BDNF-ERK signaling and the development of psychiatric comorbidity are uncertain. In this present study, we found that the expression of BDNF/ERK proteins decreased early and maintained lower after CFA injection. As its comparative effects on the psychiatric comorbidities, we also found late administration of TMP (50 mg/kg, i.p., D21-D35 for epilepsy; and 25 mg/kg, i.p., D14-D28 for chronic pain) had no effect on the hippocampus BDNF/ERK expression. Although we do not sure whether the abnormal expression of BDNF/ERK signaling was the cause, or consequence, of development of psychiatric comorbidity in late neurological disorders, but our results at least showed that early administration of TMP on neurological diseases modeling progression would be crucial for controlling the development of psychiatric comorbidity.

As above described, our present study demonstrated the inconsistent time-dependent effects of TMP on the epileptogenesis and chronic pain, compared to its effects on the psychiatric comorbidity, and elucidated a novel function for clinic TMP administration that early application of TMP might enhance hippocampal BDNF/ERK signaling to alleviate anxiety and depressive comorbidities in neurological diseases.

## Data Availability

The original contributions presented in the study are included in the article/Supplementary Material, further inquiries can be directed to the corresponding authors.

## Supplementary Material

Supplementary Figure S1 Effects of TMP on close arm entries of EPM both in Kindling and CFA induced pain models. **(A)** Effects on entries in close arms of EPM test with TMP (50 mg/kg, i.p. D21–D35 vs. D1–D35 vs. Saline) administration in after kindling. **(B)** Effects on entries in close arms of EPM test with TMP of different TMP administration periods as indicated (25 mg/kg, i.p. D0–D14, D0–D28 and D14–D28 vs. saline). Statistical analysis was performed by one way ANOVA, followed by post Dunnett’s multiple comparisons.
