# Supplementary figures and images for: Inconsistent Time-Dependent Effects of Tetramethylpyrazine on Primary Neurological Disorders and Psychiatric Comorbidities
Source: Front Pharmacol. 2021 Aug 20;12:708517. doi: 10.3389/fphar.2021.708517 (PMC8417558; doi:10.3389/fphar.2021.708517)

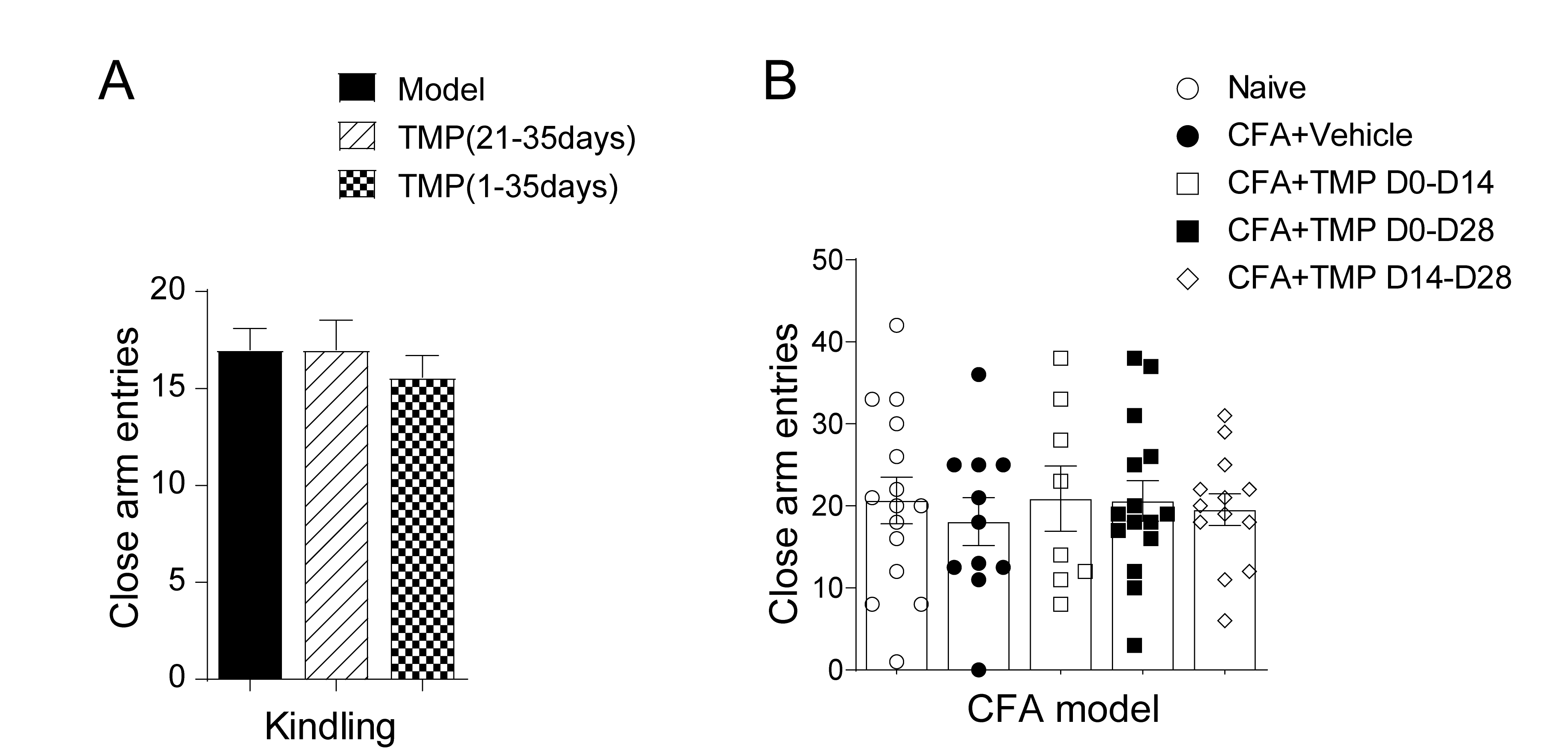

Supplement: Supplementary file 1 [file Image1.TIF]
